# Ancestral reconstruction supports that loss of Nef-mediated T cell modulation coincided with the emergence of pathogenic lentiviruses

**DOI:** 10.1128/jvi.01548-25

**Published:** 2025-12-02

**Authors:** Angelina M. Baldino, Mitchell J. Mumby, Cassandra R. Edgar, Abayomi S. Olabode, Art F. Y. Poon, Jimmy D. Dikeakos

**Affiliations:** 1 Department of Microbiology and Immunology, Western University 6221, London, Canada; 2 Department of Pathology and Laboratory Medicine, Western University 6221, London, Canada; 3 Department of Computer Science, Western University 6221, London, Canada; Icahn School of Medicine at Mount Sinai, New York, New York, USA

**Keywords:** ancestral reconstruction, Nef, simian immunodeficiency virus, human immunodeficiency virus

## Abstract

Human immunodeficiency virus type 1 (HIV-1) originated following cross-species transmission of simian immunodeficiency virus (SIV) infecting chimpanzees (SIVcpz). While SIV infection of its natural primate hosts is typically non-pathogenic, SIVcpz/HIV-1 is highly pathogenic. SIV/HIV pathogenesis is influenced by the viral accessory protein Nef, which manipulates several immune factors to facilitate disease progression. Specifically, Nef can modulate T cell activation by downregulating the T cell receptor subunit CD3ζ and the co-stimulatory receptor CD28. Several studies have demonstrated that while Nef downregulates CD28 from the T cell surface, this ability is not completely conserved throughout the SIV/HIV phylogeny. In comparison, Nef-mediated CD3 downregulation is highly conserved in SIV derived from lower-order primates yet completely absent from SIVcpz/HIV-1. Here, we used Nef proteins previously generated via an ancestral reconstruction pipeline, corresponding to common ancestors in the SIV/HIV phylogeny leading to HIV-1 group M, and evaluated their ability to downregulate CD3ζ and CD28 in both expression and transduction systems. We observed that only the ancestral Nef of all primate lentiviruses efficiently downregulated both CD3ζ and CD28, while ancestors along the SIVcpz/HIV-1 lineage failed to downregulate CD3ζ and minimally recovered the ability to downregulate CD28 over time. Furthermore, we showed that the loss of CD3ζ and CD28 downregulation was associated with increased expression of cell surface T cell activation markers and inflammatory cytokines, consistent with a pathogenic infection. Altogether, this study illustrates the evolutionary dynamics of Nef modulation of T cell activation and its impact on pathogenesis in the SIV/HIV lineage.

**IMPORTANCE:**

We characterized ancestral Nef proteins derived from the lineage that gave rise to HIV-1 group M. We show that the loss of Nef-mediated CD3ζ and CD28 downregulation occurred in the common ancestor of the SIVcpz/HIV-1 lineage and coincided with increased T cell activation, a hallmark of pathogenicity. Moreover, this suggests that evolutionary changes to Nef-mediated immune modulation contributed to the emergence of pathogenic viruses like HIV-1. Together, these findings support the application of ancestral reconstruction to better understand the functional evolution of viral proteins, particularly in the context of cross-species transmission.

## INTRODUCTION

Human immunodeficiency virus type 1 (HIV-1) was introduced to humans following multiple zoonotic transmission events, leading to four HIV-1 subgroups (M, N, O, and P), where group M (HIV-1/M) is the major causal agent of acquired immunodeficiency syndrome (AIDS) (1). HIV-1 arose following cross-species transmission events of simian immunodeficiency virus (SIV) infecting the common chimpanzee, *Pan troglodytes* (SIVcpz) (2–5). The full-length proviral sequence of SIVcpz is a genomic mosaic generated from viral recombination events. Notably, the 5′ portion, *nef* gene, and 3′ long terminal repeat resemble SIV infecting red-capped mangabeys (*Cercocebus torquatus*; SIVrcm), while the *vpu, tat, rev*, and *env* genes are most related to SIV infecting greater spot-nosed (*Cercopithecus nictitans*; SIVgsn), mustached (*Cercopithecus nictitans*; SIVmus), and mona monkeys (*Cercopithecus mona*; SIVmon) (4, 6, 7).

Serological evidence has reported endemic SIV infections in over 40 primate species (4, 8, 9). Surprisingly, early studies of SIV in sooty mangabeys (*Cercocebus atys;* SIVsmm), African green monkeys (*Chlorocebus sabaeus;* SIVagm), and mandrills (*Mandrillus sphinx*; SIVmnd) found SIV infections of these hosts to be largely non-pathogenic, characterized by low levels of inflammation and T cell activation, despite high levels of viral replication (9–12). In contrast, pathogenic infections, such as SIVcpz or HIV-1, display chronic immune activation, leading to increased rates of T cell activation, proliferation, and cell turnover within their respective hosts (13–15). These differences in pathogenicity may be explained by variations in T cell activation and inflammation. Conventional T cell activation occurs through the T cell receptor (TCR)-CD3 complex (16). The CD3ζ subunits contain intracellular immunoreceptor tyrosine-based activation motifs (ITAMs), enabling tyrosine phosphorylation and subsequent signal transduction through the TCR (16). In addition, complete T cell activation is achieved upon co-stimulatory signaling through engagement of co-stimulatory receptors like CD28 (16). Co-stimulation is required for T cell activation, as weak TCR signals may result in suppression of the immune response and anergy (16). Together, T cell activation requires specific signaling cascades via motifs on the CD3ζ subunit of the TCR and co-stimulatory signals from CD28 (16).

To facilitate successful infection, SIV and HIV rely on accessory proteins to counteract host immune responses *in vivo*. In particular, the Nef accessory protein has been implicated in viral pathogenicity by manipulating the host immune responses to promote immune evasion (17, 18). For example, Nef downregulates CD4 and major histocompatibility complex class I (MHC-I) from the cell surface to evade virus detection by the adaptive immune response (19, 20). Nef can also counter aspects of the host’s intrinsic innate immune response by downregulating serine incorporator 5 (SERINC5) and, to a lesser extent, SERINC3, to enhance viral infectivity (21). Numerous studies have also shown that Nef can alter T cell responses, which may explain differences observed in the pathogenicity of SIV and HIV infections. Specifically, Nef downregulates the CD28 co-stimulatory receptor from the T cell surface, which can be inhibited upon mutation of the Nef dileucine (LL_165_) and diacidic (DD_175_) motifs (22–24). Moreover, SIV Nefs from the lineage that gave rise to HIV-2 downregulate CD28 more efficiently than Nefs from SIVcpz/HIV-1 and SIVmus/mon/gsn (22). Therefore, Nef-mediated CD28 downregulation is not completely conserved and functionally varies throughout the SIV/HIV phylogeny.

Nef also downregulates CD3 via interactions with the CD3ζ subunit, hereafter referred to as CD3 (22, 25–30). While this function is highly conserved in SIV derived from lower-order primates, Nef proteins derived from the HIV-1 lineage—HIV-1, SIVcpz, SIVmus/mon/gsn—fail to downregulate CD3 altogether (22, 25, 26, 29, 30). The Nefs that downregulate CD3 efficiently are derived from SIV infections that are non-pathogenic in their natural hosts (22). Since pathogenicity may be related to T cell activation, it is unsurprising that pathogenic infections correlate with the inability of Nef to downregulate CD3. Furthermore, Nefs derived from SIVcpz/HIV-1 and SIVmus/mon/gsn—which do not downregulate CD3—display increased expression of T cell activation markers (22, 26, 30). Overall, the ability of SIV Nef to modulate T cell activation by downregulating CD3 and CD28 is consistent with a non-pathogenic phenotype, while retaining these receptors on the T cell surface leads to increased T cell activation and inflammation, consistent with a pathogenic infection. Overall, the virus is not the only essential component for determining pathogenicity; instead, it is a summation of both virus- and host-specific adaptations (22, 31).

Prior studies seeking to understand the evolution of Nef as a pathogenic factor have traditionally utilized modern-day—or extant*—nef* sequences (reviewed in reference 32). This approach can limit information regarding the evolutionary dynamics of Nef within a phylogeny. We previously employed an ancestral reconstruction pipeline to generate an accurate time-scaled phylogeny relating Nef sequences from both SIV and HIV lineages. This approach predicted the ancestral Nef sequences at each common ancestor, or node, in the phylogenetic lineage leading to HIV-1 group M and generated corresponding nucleotide sequences (33). Thus far, the reconstructed ancestral Nefs have exclusively been tested for their abilities to downregulate SERINC5 and CD4 (33). The investigation of Nef functions pertaining to T cell activation, such as CD3 and CD28 downregulation, has not been assessed.

Herein, we further investigated the reconstructed phylogeny by assessing the ability of these predicted Nefs to downregulate CD3 and CD28—two functions that likely contribute to pathogenicity by modulating T cell responses. We found that the Nef corresponding to the ancestor of all primate lentiviruses efficiently downregulated CD3 and CD28, while the ancestors derived from SIVcpz/HIV-1 failed to do so. Furthermore, we observed that robust CD3 and CD28 downregulation led to low levels of T cell activation, consistent with a non-pathogenic infection. In comparison, expression of Nefs incapable of CD3 and CD28 downregulation led to high levels of T cell activation, consistent with the pathogenic nature of SIVcpz/HIV-1. This characterization of Nef ancestors provides insight into how Nef-mediated CD3 and CD28 downregulation may have evolved through time and helps create predictions for how these changes contributed to the pathogenic nature of SIVcpz and HIV-1 infection within their respective hosts.

## RESULTS

### Ancestral Node 35 Nef efficiently downregulates cell-surface CD3, independent of host species

**Fig 1:**
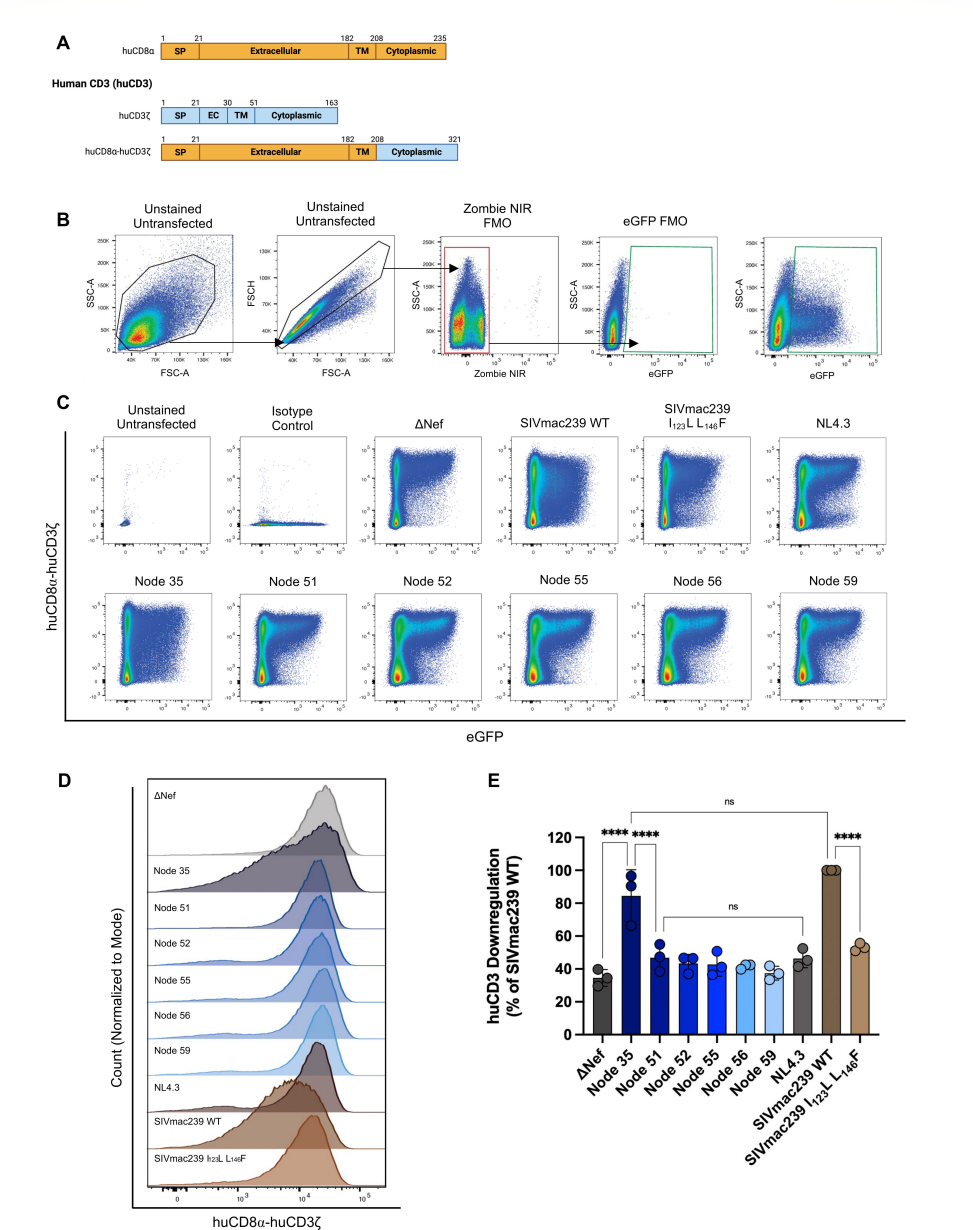
Ancestral Nef downregulation of cell surface human CD3 (huCD3). HEK293T cells were triply transfected with pNL4.3 ΔGag/Pol eGFP ΔVpu Nef plasmids, the pDR8.2 plasmid encoding Gag/Pol, and a plasmid encoding a huCD8α-huCD3ζ fusion construct. Cell surface levels of huCD8α were measured as a proxy for Nef-mediated huCD3 downregulation 48 hours post-transfection via flow cytometry. (**A**) Schematic depicting the topological domains of huCD8α (top; orange), huCD3ζ (middle; blue), and huCD8α-huCD3ζ fusion construct (bottom). (**B**) Gating strategy used to isolate live (Zombie NIR^-^) and transfected (eGFP^+^) cells. (**C**) Representative pseudocolor plots illustrating huCD8α-huCD3ζ (PE; *Y*-axis) and eGFP (*X*-axis) levels gated on live (Zombie NIR^-^) cells. (**D**) Representative histogram of cell surface levels of huCD8α-huCD3ζ (*X*-axis) within live, transfected (Zombie NIR^-^, eGFP^+^) cells. The count is normalized to the mode of the set. (**E**) Summary of fold huCD3 downregulation (±SE) from three independent experiments (*n* = 3), calculated as the percentage of SIVmac239 WT Nef (positive control). SP, signal peptide; EC, extracellular domain; TM, transmembrane domain; SSC-A, side scatter area; FSC-A, forward scatter area; FSC-H, forward scatter height; FMO, fluorescence minus one; NIR, near-infrared; eGFP, enhanced green fluorescent protein; PE, phycoerythrin; and SE, standard error. Significance: ****P < 0.0001; ns, not significant. Schematic created in BioRender. https://BioRender.com/p20gixl.

The reconstructed phylogeny contains six internal nodes in the path from the ancestral primate lentivirus to the common ancestor of HIV-1/M (33). The ancestral nodes (Fig. S1) correspond to the following lineages: Node 35 (all primate lentiviruses, including SIVcpz, SIVrcm, SIVsun, SIVmus/mon/gsn, HIV-1, and HIV-2), Node 51 (HIV-1/SIVsun), Node 52 (HIV-1/SIVcpz), Node 55 (HIV-1/SIVcpzptt), Node 56 (HIV-1/M,N/SIVcpzptt), and Node 59 (HIV-1/M). We first tested the ability of the reconstructed Nefs to downregulate cell surface CD3. To do so, we expressed the various Nefs in HEK293T cells and used the huCD8α-CD3ζ fusion constructs (Fig. 1A) to evaluate the downregulation of CD3 sequences derived from different primate hosts, including human/chimpanzee (huCD3), which has been evaluated previously (30). We also included a Nef isolate derived from a well-characterized strain of SIV—SIVmac239—which has been previously shown to robustly downregulate both huCD3 and rhesus macaque CD3 (rhCD3) (22, 25, 30). As a functional negative control, we included a mutated form of SIVmac239 Nef containing two mutations in its core domain, I_123_L L_146_F, that selectively impair CD3 downregulation (25). We also included a lab-adapted HIV-1 Nef (NL4.3 Nef), which has also been shown to be incapable of CD3 downregulation (22, 25, 30). Finally, we included a plasmid that expresses eGFP only (ΔNef) as our overall negative control. Accordingly, we assessed the ability of the various Nefs to downregulate huCD3. As expected, no huCD3 downregulation was observed in the ΔNef and NL4.3 Nef negative controls (Fig. 1C through E; 34.5% and 46.4% of SIVmac239 WT, respectively, *P* < 0.0001). Additionally, SIVmac239 WT Nef demonstrated robust huCD3 downregulation, while its mutant form was significantly impaired, as expected (Fig. 1C through E; 53.2% of SIVmac239 WT, *P* < 0.0001). We observed that Node 35 Nef, which represents the ancestral primate lentivirus, displayed a significant increase in huCD3 downregulation compared to the ΔNef negative control (Fig. 1C through E; 84.4% of SIVmac239 WT, *P* < 0.0001). Furthermore, Node 35 Nef downregulated huCD3 to a significantly greater extent when compared to the rest of the ancestral Nefs (Fig. 1C through E; *P* < 0.0001), which displayed a similar level of downregulation as ΔNef (Fig. 1C through E; ranging from 37.5% to 46.9% of SIVmac239 WT). To compare the relative expression levels of the ancestral Nef proteins, the codon-optimized *nef* sequences were cloned into an expression vector enabling C-terminal eGFP fusion, as described previously (33). The resulting constructs were transfected into HEK293T cells, and Nef-eGFP expression levels were evaluated by Western blot analysis (Fig. S2). Although the expression levels varied slightly among the reconstructed Nef-eGFP variants, all fusion proteins were expressed at high levels (Fig. S2). Together, these findings provide evidence that the ancestral reconstruction accurately generated Nef ancestors, given that all SIVcpz/HIV-1 Nefs were incapable of CD3 downregulation, as expected.

**Fig 2:**
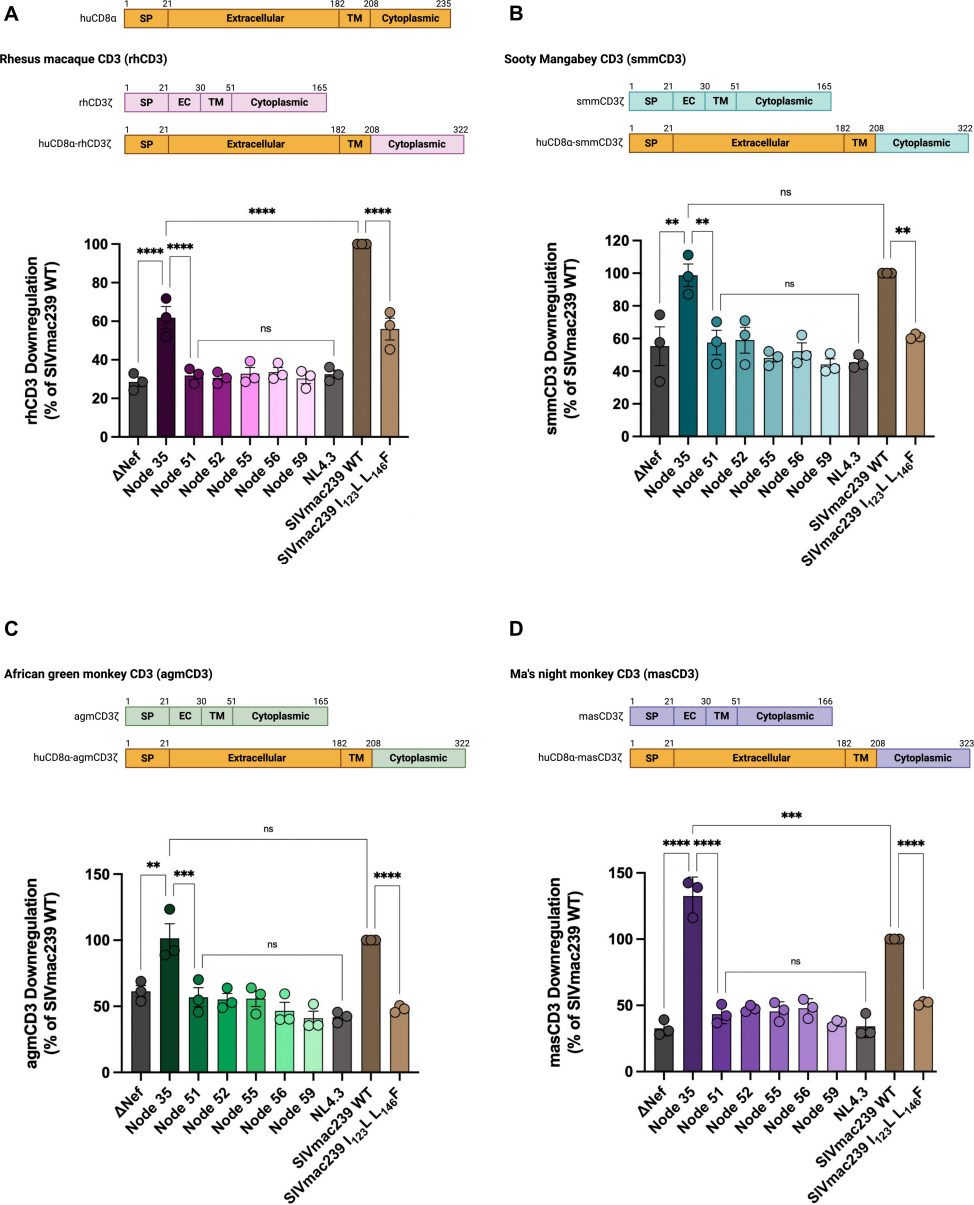
Ancestral Nef downregulation of cell surface non-human primate CD3. Cell surface levels of huCD8α were measured as a proxy for Nef-mediated downregulation of (**A**) rhesus macaque CD3 (rhCD3), (**B**) sooty mangabey CD3 (smmCD3), (**C**) African green monkey CD3 (agmCD3), and (**D**) Ma’s night monkey CD3 (masCD3). (A–D) Top: schematic depicting the topological domains of huCD8α (orange) fused to the cytoplasmic domain of each non-human primate CD3ζ to form a huCD8α-CD3ζ fusion construct. (**A–D**) Bottom: summary of fold CD3 downregulation (±SE) within live, transfected (Zombie NIR^-^, eGFP^+^) cells. Data from three independent experiments (*n* = 3), calculated as the percentage of SIVmac239 WT Nef. SP, signal peptide; EC, extracellular domain; TM, transmembrane domain; NIR, near-infrared; eGFP, enhanced green fluorescent protein; SE, standard error. Significance: **P < 0.01; ***P < 0.001; and ****P < 0.0001; ns, not significant. Schematic created in BioRender. https://BioRender.com/p20gixl.

We next tested if Nef-mediated CD3 downregulation differs within various species contexts, as some Nef functions are species-dependent. For instance, SIV Nefs can antagonize their autologous tetherin, a host factor that interferes with virion release, but not human tetherin (34–36). Contrastingly, some Nef functions, such as SERINC5 downregulation, are independent of the host species, with SIV and HIV Nefs retaining the ability to downregulate SERINC5 homologs across different primates (30, 33, 37, 38). Thus, we generated additional huCD8α-CD3ζ fusions encoding the CD3ζ cytoplasmic tail of rhesus macaques (rhCD3; Fig. 2A), sooty mangabeys (smmCD3; Fig. 2B), African green monkeys (agmCD3; Fig. 2C), and Ma’s night monkeys (masCD3; Fig. 2D). Given that sooty mangabeys and African green monkeys are naturally infected with SIV, we expected to see a similar trend to huCD3 downregulation, where HIV-1 Nefs are incapable of downregulating CD3. On the other hand, Ma’s night monkey is a new world primate and is not naturally infected with SIV. However, recent evidence suggests that these primates contain compatible CD4 receptors for HIV-1 and may serve as a novel non-human primate model for studying pathogenic HIV-1 infections (39, 40). Therefore, we expected the pattern of masCD3 downregulation to emulate our previous experiments where only Node 35 Nef could downregulate CD3.

The evaluation of rhCD3, smmCD3, agmCD3, and masCD3 downregulation demonstrated that Node 35 Nef displayed significant CD3 downregulation (Fig. 2A through D; 61.9%, 98.8%, 101.5%, and 132.5% of SIVmac239 WT, respectively; ranging from *P* < 0.01 to *P* < 0.0001), but Node 51-59 Nefs did not. In these experiments, we observed no significant difference in CD3 downregulation between Node 35 Nef and SIVmac239 WT Nef (Fig. 2B and C; *P* > 0.05), except in the context of rhCD3 downregulation, where SIVmac239 WT Nef demonstrated significantly greater CD3 downregulation than Node 35 Nef (Fig. 2A; *P* < 0.0001). Interestingly, when evaluating masCD3 downregulation, Node 35 Nef displayed significantly greater masCD3 downregulation than SIVmac239 WT Nef (Fig. 2D; *P* < 0.0001). This was contrary to all previous experiments in which Node 35 Nef exhibited significantly less (rhCD3) or similar (smmCD3 agmCD3) CD3 downregulation to SIVmac239 WT Nef. Taken together, the Nef representing the ancestor of all primate lentiviruses (Node 35) displayed significant CD3 downregulation, regardless of species context. In comparison, Nefs derived from the SIVcpz/HIV-1 lineage (Nodes 51–59) failed to downregulate CD3 across different species contexts. Therefore, this function appears independent of the host species, suggesting that downstream experiments in primary human cells can be generalizable across most primates.

### Ancestral Node 35 Nef efficiently downregulates cell-surface CD3 and CD28 in transduced primary CD4^+^ T cells

**Fig 3:**
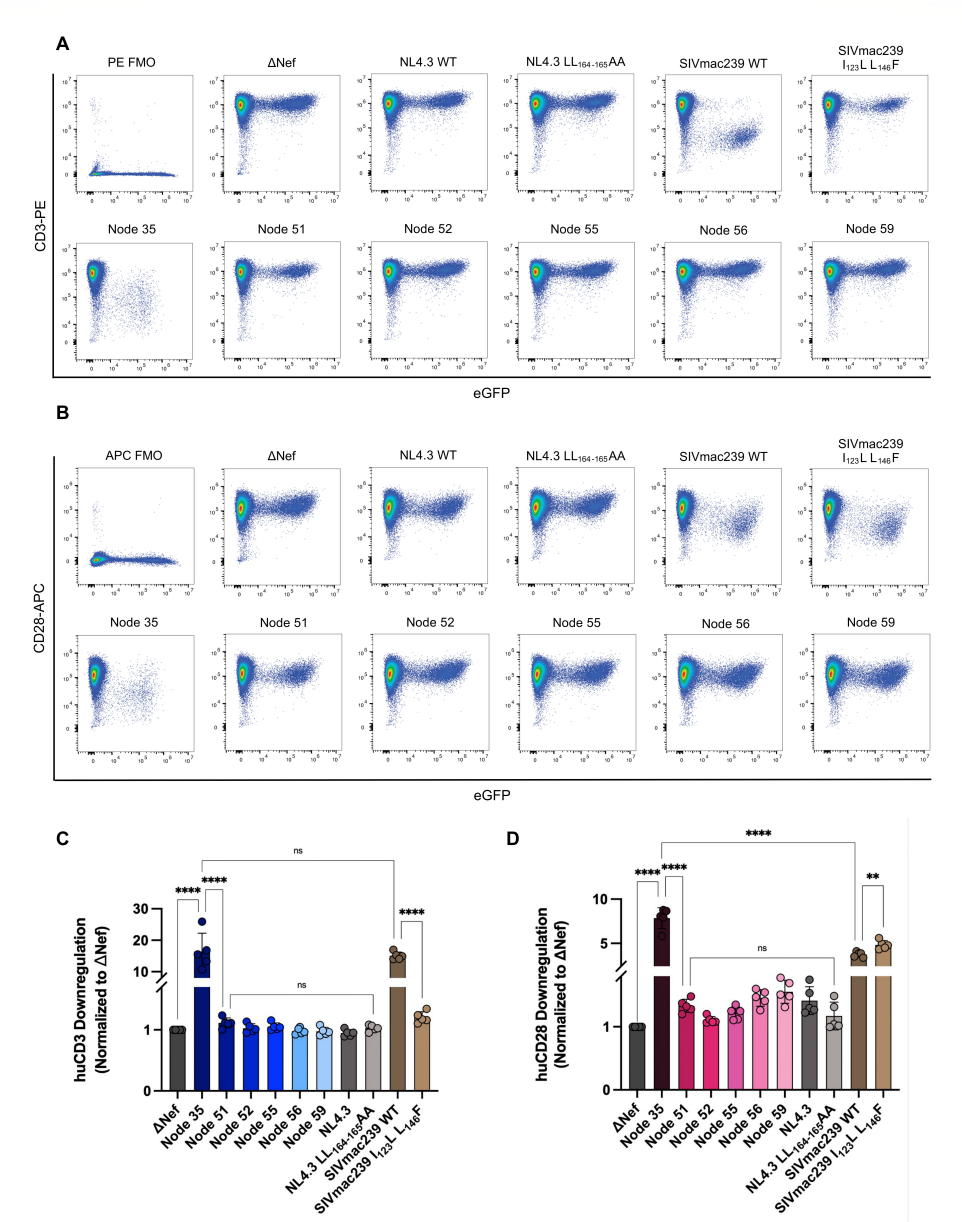
Ancestral Nef downregulation of cell surface CD3 and CD28 in transduced isolated primary huCD4^+^ T cells. Activated huCD4^+^ T cells were transduced with replication-incompetent, VSV-G-pseudotyped viruses via spinoculation. Cell surface levels of CD3 and CD28 were measured 48 hours post-transduction via flow cytometry. (**A**) Representative pseudocolor plots illustrating CD3 (PE; *Y*-axis) and eGFP (*X*-axis) levels gated on live (Zombie NIR^-^) cells. (**B**) Representative pseudocolor plots illustrating CD28 (APC; *Y*-axis) and eGFP (*X*-axis) levels gated on live (Zombie NIR^-^) cells. Summary of fold (**C**) CD3 and (**D**) CD28 downregulation (±SE) within live, transduced (Zombie NIR^-^, eGFP^+^) cells. Data collected from the transduction of five primary cell donors (*n* = 5), normalized to ΔNef (negative control). NIR, near-infrared; eGFP, enhanced green fluorescent protein; PE, phycoerythrin; APC, allophycocyanin; and SE, standard error. Significance: **P < 0.01; ****P < 0.0001; ns, not significant.

We next tested the function of the various Nef proteins in primary human CD4^+^ (huCD4^+^) T cells transduced with pseudoviruses expressing each ancestral Nef. In parallel, we also assessed the ability of these Nefs to downregulate CD28. Of note, the CD28 cytoplasmic amino acid sequences are identical between humans, chimpanzees, rhesus macaques, sooty mangabeys, and African green monkeys, but not with Ma’s night monkeys. Given the highly conserved nature of the CD28 sequences, we predicted this Nef function to, like CD3 downregulation, also be species independent. We included the same controls described previously, with the addition of a mutated NL4.3 Nef (NL4.3 LL_164-165_AA Nef), containing a mutated dileucine motif, which prevents the interaction between Nef and AP-2 to impair CD28 downregulation (24). While trends of CD3 downregulation observed in transduced primary huCD4^+^ T cells were consistent with Fig. 1C through E, the magnitude of Nef-mediated CD3 downregulation was considerably more pronounced upon transduction (Fig. 3A and C). Specifically, Node 35 Nef demonstrated significantly greater CD3 downregulation than ΔNef (Fig. 3C; 17.6-fold, *P* < 0.0001), while NL4.3 and Nodes 51–59 Nefs did not differ from ΔNef (Fig. 3C; ranging from 0.95- to 1.1-fold). Moreover, SIVmac239 WT Nef significantly downregulated CD3 compared to ΔNef (Fig. 3C; 13.8-fold, *P* < 0.0001), while the mutant SIVmac239 I_123_L L_146_F Nef was significantly impaired compared to SIVmac239 WT Nef (Fig. 3C; 1.2-fold, *P* < 0.0001). Finally, within transduced primary cells, there was no significant difference in huCD3 downregulation between Node 35 Nef and SIVmac239 WT Nef (Fig. 3C; *P* > 0.05).

The pattern of Nef-mediated CD28 downregulation differed slightly from CD3 downregulation (Fig. 3B and D). Both SIVmac239 WT Nef and SIVmac239 I_123_L L_146_F Nef downregulated CD28 (Fig. 3D; 3.7- and 4.8-fold, respectively), confirming that the I_123_L L_146_F mutation selectively disrupts CD3 downregulation, although this mutation led to a significant increase in CD28 downregulation compared to SIVmac239 WT Nef (Fig. 3D; *P* < 0.01). As expected, Node 35 Nef significantly downregulated CD28 compared to ΔNef (Fig. 3D; 3.5-fold, *P* < 0.0001). However, unlike CD3 downregulation, we observed differential CD28 downregulation with Nodes 51–59. For instance, we found Nodes 51 and 52 Nef downregulated CD28 significantly less than Node 35 Nef (Fig. 3D; 1.3- and 1.1-fold, respectively, *P* < 0.0001). Interestingly, we observed that CD28 downregulation gradually improved in Nodes 55 (1.2-fold), 56 (1.5-fold), and 59 Nefs (1.6-fold). Thus, CD28 downregulation is lost from Node 35 to Node 52 and then gradually recovered in Nodes 55–59. These findings are contrary to CD3 downregulation, which is completely lost in the SIVcpz/HIV-1 lineage (Nodes 51–59), suggesting the evolutionary dynamics of CD28 downregulation mediated by Nef differs from CD3 downregulation.

### Efficient CD3 and CD28 downregulation significantly impairs T cell activation

**Fig 4:**
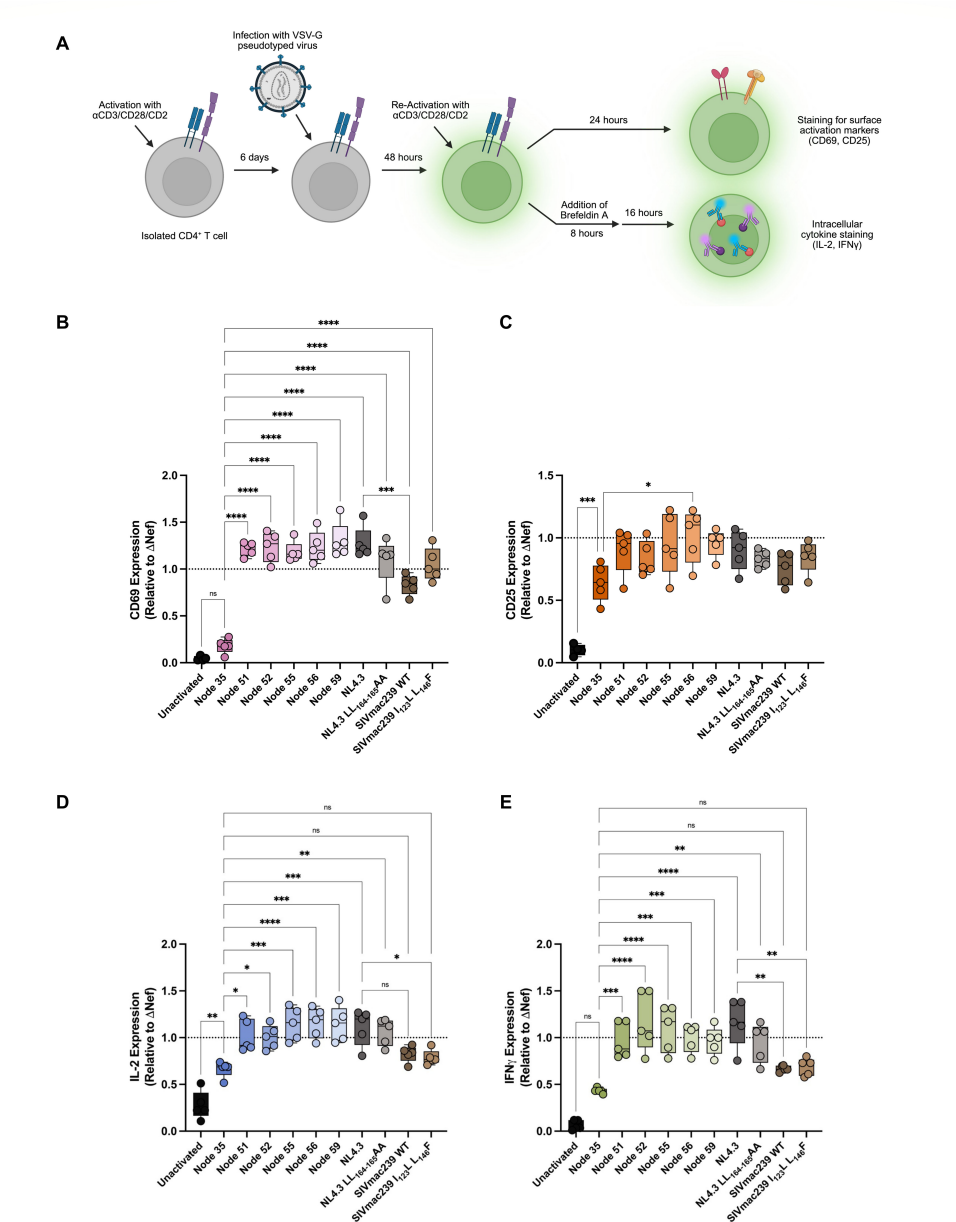
Downstream consequences of Nef-mediated cell surface CD3 and CD28 downregulation on T cell activation and intracellular cytokine production. (**A**) Schematic depicting the experimental workflow. Isolated huCD4^+^ T cells were activated upon thawing with Immunocult human CD3/CD28/CD2 T cell activator and expanded for 6 days before transduction with replication-incompetent, VSV-G-pseudotyped viruses via spinoculation. Forty-eight hours post-transduction, the cells were reactivated. Cell surface levels of CD69 and CD25 were measured 24 hours post-re-activation via flow cytometry. Intracellular levels of IL-2 and IFNγ were measured 24 hours post-re-activation via flow cytometry, with the addition of Brefeldin A 8 hours post-re-activation to stop cytokine secretion. Summary of cell surface expression of (**B**) CD69 and (**C**) CD25 (±SE) gated on live, transduced (Zombie NIR^-^, eGFP^+^) cells. Data collected from transduction of five primary cell donors (*n* ≥ 4) and normalized to ΔNef (represented by the dotted line). Unactivated control is transduced with ΔNef. Summary of intracellular expression of (**D**) IL-2 and (**E**) IFNγ (±SE) gated on live, transduced (Zombie NIR^-^, eGFP^+^) cells. Data collected from transduction of five primary cell donors (*n* = 5), normalized to ΔNef (represented by the dotted line). NIR, near-infrared; eGFP, enhanced green fluorescent protein; PE, phycoerythrin; AF647, AlexaFluor647; and SE, standard error. Significance: *P < 0.05; **P < 0.01; ***P < 0.001; and ****P < 0.0001; ns, not significant. Schematic created in BioRender. https://BioRender.com/p20gixl.

We next evaluated the downstream effects of Nef-mediated CD3 and/or CD28 downregulation on T cell activation following transduction. To do this, we transduced primary huCD4^+^ T cells with the pseudoviruses expressing each ancestral Nef. Following transduction, T cells were re-stimulated via the TCR using a soluble anti-CD3/CD28/CD2 antibody complex. We then measured the expression of surface activation markers CD69 and CD25 and the intracellular production of pro-inflammatory cytokines IL-2 and IFNγ (Fig. 4A). We hypothesized that the expression of Nefs capable of robust CD3 and/or CD28 downregulation would lead to lower T cell activation, as efficient downregulation of CD3 and/or CD28 would render transduced cells less able to become activated upon the addition of a secondary stimulus. As expected, in the presence of Node 35 Nef, we observed a significant decrease in CD69 expression compared to all ancestral and control Nefs (Fig. 4B; *P* < 0.0001), consistent with its ability to efficiently downregulate both CD3 and CD28. This was also not significantly different than the unactivated control, which was transduced with ΔNef (Fig. 4B; *P* > 0.05). SIVmac239 WT Nef, which also robustly downregulates CD3 and CD28, displayed an intermediate activation profile, with CD69 expression levels significantly greater than Node 35, but significantly less than HIV-1 Nefs such as NL4.3 WT Nef (Fig. 4B; *P* < 0.05). In the context of CD25, we observed cells expressing Node 35 Nef retained less CD25 on the cell surface when compared to Nodes 51–59, although these were not significantly different (Fig. 4C; *P* > 0.05). Similarly, when we assessed the expression of IL-2 and IFNγ, we observed Node 35 Nef to result in significantly less intracellular IL-2 and IFNγ production than all the ancestral Nefs (Fig. 4D and E; ranging from *P* < 0.05 to *P* < 0.0001). This did not differ from the cytokine expression levels in the presence of SIVmac239 Nef (WT or mutant), which were also significantly less than NL4.3 WT Nef (Fig. 4D and E; *P* < 0.05). In conclusion, T cell activation in the presence of the reconstructed Nefs generally aligns with the magnitude by which each viral protein downregulates CD3 and/or CD28 from the cell surface.

### Mapping regions involved in CD3 and CD28 downregulation by Node 35 Nef

**Fig 5:**
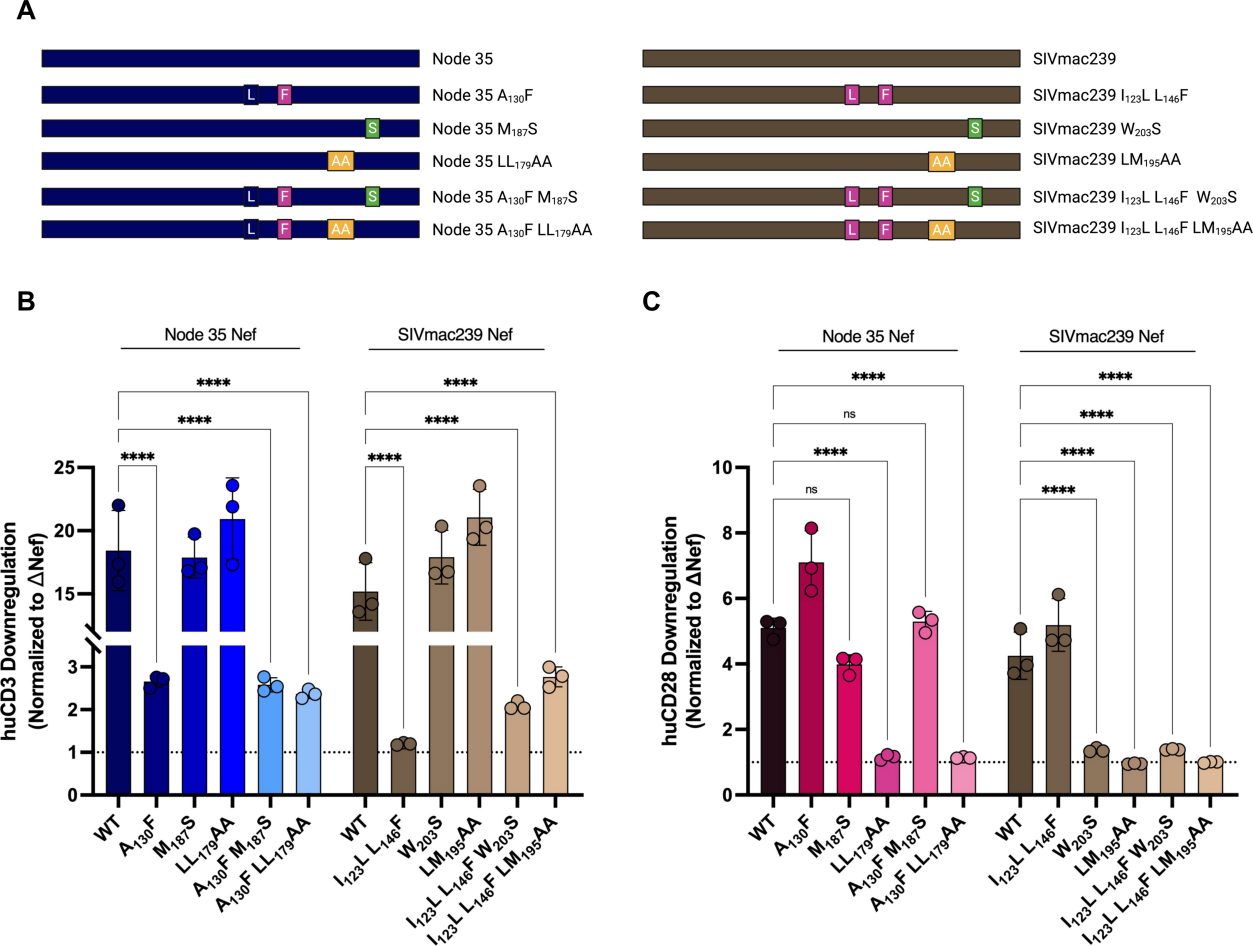
Mapping regions involved in CD3 and CD28 downregulation by Node 35 and SIVmac239 Nef. Activated huCD4^+^ T cells were transduced with replication-incompetent, VSV-G-pseudotyped viruses via spinoculation. Cell surface levels of CD3 and CD28 were measured 48 hours post-transduction via flow cytometry. (**A**) Schematic depicting Node 35 and SIVmac239 Nef mutants. Summary of fold (**B**) CD3 and (**C**) CD28 downregulation (±SE) within live, transduced (Zombie NIR^-^, eGFP^+^) cells. Data collected from transduction of three primary cell donors (*n* = 3), normalized to ΔNef (negative control). NIR, near-infrared; eGFP, enhanced green fluorescent protein; and SE, standard error. Significance: ****P < 0.0001; ns, not significant. Schematic created in BioRender. https://BioRender.com/p20gixl.

To elucidate the motifs involved in CD3 and CD28 downregulation by Node 35 Nef, we first aligned its amino acid sequence to SIVmac239 Nef, which shares these functional capabilities, and identified putative motifs involved in CD3 or CD28 downregulation using sequence homology (Fig. S3A). We then generated the corresponding mutations in Node 35 Nef that would selectively impair CD3 or CD28 downregulation without affecting the downregulation of the other molecule. To assess CD3 downregulation, we generated the Node 35 A_130_F mutant, mirroring the I_123_L L_146_F mutation in SIVmac239 Nef, which we (Fig. 1E, 2, and 3C) and others have demonstrated to selectively ablate CD3 downregulation without affecting CD28 downregulation (25). Moreover, the W_203_S mutation in SIVsmm Nef—corresponding to the same residue in SIVmac239 Nef (W_203_S)—can significantly impair CD28 downregulation, but not CD3 downregulation (41). In addition, the dileucine motif of SIVmac239 Nef (LM_194-195_) is essential for CD28 downregulation, but mutation of this motif (LM_194-195_AA) does not impact CD3 downregulation (42, 43). Considering this, we generated two mutants to selectively impair CD28 downregulation in Node 35 Nef: M_187_S and LL_178-179_AA. In addition, we combined these mutations (M_187_S and LL_178-179_AA) to generate Nef proteins predicted to be incapable of downregulating both CD3 and CD28 (Node 35 A_130_F M_187_S or Node 35 A_130_F LL_178-179_AA and SIVmac239 I_123_L L_146_F W_203_S or SIVmac239 I_123_L L_146_F LM_194-195_AA; Fig. 5A).

We next investigated the ability of the various Node 35 and SIVmac239 Nef mutants to downregulate CD3 and CD28 in transduced huCD4^+^ T cells. As in previous experiments, both Node 35 and SIVmac239 Nef demonstrated robust CD3 (18.4- and 15.2-fold, respectively; Fig. 5B and C) and CD28 downregulation (5.1- and 4.3-fold, respectively; Fig. 5B and C). In comparison, the I_123_L L_146_F mutation in SIVmac239 and the corresponding A_130_F mutation in Node 35 Nef significantly impaired CD3 downregulation (2.7- and 1.2-fold, respectively, *P* < 0.0001), but not CD28 downregulation (Fig. 5B and C). As expected, both the W_203_S and LM_195_AA mutations in SIVmac239 Nef significantly impaired CD28 downregulation (1.4- and 0.95-fold, respectively, *P* < 0.0001), but not CD3 downregulation (Fig. 5B and C). However, while the corresponding dileucine motif mutation in Node 35 Nef (LL_178-179_AA) significantly diminished CD28 downregulation (1.2-fold, *P* < 0.0001), but not CD3 downregulation, the M_187_S mutation only slightly impacted CD28 downregulation in Node 35 Nef (4-fold, *P* > 0.05). Additionally, we evaluated the relative expression levels of the mutant Node 35 and SIVmac239 Nef proteins by Western blot analysis (Fig. S3B) and observed similar expression levels of all Nef proteins. These findings suggest that Node 35 and SIVmac239 Nef rely on similar residues to facilitate CD3 downregulation (A_130_ and I_123_ L_146_, respectively) and on the dileucine motif (LL_178-179_ and LM_194-195_, respectively) to selectively downregulate CD28. However, the observation that the M_187_S mutation in Node 35 Nef fails to significantly inhibit CD28 downregulation suggests that this reconstructed Nef is structurally and/or functionally distinct from SIVmac239 Nef and may rely on other motifs to exert its functions.

## DISCUSSION

In the current report, we assessed the ability of reconstructed ancestral Nef proteins to downregulate cell-surface CD3 and CD28. Functional outputs were also used to further validate the previously employed bioinformatics pipeline (33). Overall, these analyses allowed us to characterize the evolutionary changes of Nef functions and determine the contribution of these Nef-mediated functions to pathogenicity.

We determined that only the ancestor of all primate lentiviruses, Node 35 Nef, efficiently downregulated cell surface CD3. This function was subsequently lost in the SIVcpz/HIV-1 lineage and never recovered, regardless of the host primate context. These findings align with previous reports suggesting that Nef-mediated CD3 downregulation is only retained in the majority of SIV and HIV-2 Nefs, while absent in the SIVcpz/HIV-1 lineage (22, 26, 30). Although we determined that Nef-mediated CD3 downregulation is independent of the host species, we observed some divergence in its host-specific magnitude. For instance, SIVmac239 WT Nef was less efficient than Node 35 Nef at downregulating masCD3, which may implicate the role of host-virus co-evolution. We posit that SIVmac239 Nef evolved robust rhCD3 downregulation following extensive co-evolution, which came at the consequence of modestly impairing its ability to downregulate CD3 from an unrelated primate species, such as Ma’s night monkeys. On the contrary, smmCD3 and agmCD3 are more genetically similar to rhCD3 than to masCD3, explaining why we observed a similar pattern of CD3 downregulation between these experiments. Additionally, these findings may provide further insight into the potential for Ma’s night monkeys to serve as a novel model for studying HIV-1 infections. The results from the evaluation of masCD3 downregulation align with what would be expected for a non-human primate model of HIV-1 infection, where CD3 downregulation is not observed in the presence of HIV-1 Nefs—like NL4.3 Nef—but does occur in the presence of other SIV Nefs such as SIVmac239 Nef (Fig. 2D).

In addition, Node 35 Nef was the only ancestral Nef capable of robust CD28 downregulation. Similar to Nef-mediated CD3 downregulation, this function was subsequently lost in Nodes 51 and 52. The magnitude of CD28 downregulation by Node 59 Nef, which represents the ancestor of HIV-1 group M, is similar to the lab-adapted HIV-1 NL4.3 Nef, which aligns with reports suggesting that HIV-1 Nefs exhibit weak CD28 downregulation that is not as efficient as SIV and HIV-2 Nefs (22, 24, 30, 42).

We then examined the downstream consequences of Nef-mediated CD3 and CD28 downregulation on T cell activation within transduced cells. Given the magnitude of CD3 and CD28 downregulation we observed previously, we expected transduced cells expressing SIVmac239 WT Nef to display similar levels of T cell activation to Node 35. The finding that SIVmac239 WT Nef led to an intermediate activation profile in primary human cells may suggest that a specific host factor in rhesus macaques is required for maintaining lower immune activation in the presence of a Nef isolate that downregulates CD3 and CD28. In addition, other studies that observed low levels of T cell activation (i.e., CD69 expression) in the presence of SIVmac239 WT Nef used replication-competent virus and infected peripheral blood mononuclear cells (PBMCs) (22, 30). For our study, we utilized a replication-incompetent virus and transduced purified primary CD4^+^ T cells to limit the potential confounding effects of other immune cells in the population. Altogether, this may explain why we did not observe the same activation profile between Node 35 and SIVmac239 Nef.

Our finding that efficient Nef-mediated CD3 downregulation by Node 35 Nef led to lower levels of immune activation is consistent with the non-pathogenic phenotype of SIV infections within their respective natural hosts. When this function is lost, we observed greater T cell responses, consistent with a pathogenic infection. However, it remains unclear why Nef would lose its ability to downregulate CD3, given its role in reducing the magnitude of the host immune response. It has been hypothesized that retaining CD3 on the surface of infected T cells may provide a selective advantage for the virus, as greater T cell signaling increases T cell recruitment and generates larger pools of T cells susceptible to viral infection (25). An increase in T cell responsiveness can also boost viral gene expression and replication (26). Additionally, cell surface CD3 expression enhances Env incorporation into virions, thereby increasing viral infectivity and spread (26). This aligns with our findings that the loss of Nef-mediated CD3 downregulation in the SIVcpz/HIV-1 group M lineage is associated with features of a pathogenic infection, such as the increased expression of T cell activation markers and inflammatory cytokines.

We have also provided insight into how motifs in Node 35 Nef may facilitate CD3 and CD28 downregulation by mapping the corresponding residues and functional motifs observed with SIVmac239 Nef using sequence homology. The finding that the SIVmac239 I_123_L L_146_F and Node 35 A_130_F mutants selectively impaired CD3 downregulation but not CD28 downregulation suggests that these Nefs rely on similar residues within the core Nef domain to associate with and downregulate CD3. Similarly, mutation of the Nef dileucine motif (SIVmac239 LM_194-195_AA and Node 35 LL_178-179_AA) significantly impaired CD28 downregulation, while not affecting CD3 downregulation, suggesting that the dileucine motif is required for CD28 downregulation to facilitate Nef and AP-2 binding (41). Interestingly, while the W_203_S mutation in SIVmac239 Nef demonstrated a similar outcome as the LM_195_AA mutant, the corresponding mutation in Node 35 Nef (M_187_S) did not. Previously, the W_203_S mutation in SIVsmm Nef was shown to disrupt CD28 downregulation—but not CD3 downregulation—by impacting Nef complex formation with AP-2 subunits (41). We speculate that Node 35 Nef and SIVmac239 Nef exhibit similar, but distinct, mechanisms to downregulate CD28 via AP-2 engagement. Both Nef proteins require binding of their dileucine motif to AP-2, but rely on alternate residues to stabilize the interactions within the Nef ternary complex in the context of CD28 downregulation.

The loss of Nef-mediated CD3 downregulation in SIVcpz/HIV-1 coincides with the acquisition of another viral protein, Vpu (22). Specifically, HIV-1, SIVcpz, and the SIVmus/mon/gsn clade encode *vpu*, while other SIVs and HIV-2 encode a *vpx* gene (44, 45). This suggests that Vpu originated from the common ancestor of SIVmus/mon/gsn and was then transmitted to SIVcpz following its recombination with SIVrcm (6, 45). The acquisition of *vpu* by SIVcpz through recombination was considered necessary for its cross-species transmission to humans, as the Nef protein in SIVcpz/HIV-1 lost the ability to downregulate human tetherin, a function that was regained by Vpu (46). This is especially interesting because Nef proteins derived from the SIVmus/mon/gsn clade were previously found to lack the ability to downregulate CD3, despite representing an SIV lineage (22). However, Vpu does not directly impact T cell activation via the downregulation of CD3. More specifically, Nef-mediated CD3 downregulation prevents the induction of NF-kB, while HIV-1 Vpu inhibits NF-kB signaling at later steps in the pathway to prevent antiviral gene expression (47–49). This suggests that Vpu-mediated suppression of T cell activation downstream in the NF-kB pathway may have reduced the selective pressure for Nef to suppress T cell activation by downregulating CD3 (47–49). For the current study, we used a proviral backbone lacking Vpu to ensure that our findings were not the result of overlapping functions between the two viral proteins. Nonetheless, the evolution of Nef-mediated CD3 downregulation coincided with the acquisition of Vpu, thereby providing the virus with an alternative method of immune suppression while maintaining the benefits of increased T cell signaling through the TCR.

While the magnitude of Nef-mediated CD3 downregulation likely drives the downstream impacts on T cell activation, we also observed robust CD28 downregulation by Nefs that efficiently downregulated CD3 (such as Node 35 and SIVmac239 WT Nef). These Nefs may robustly downregulate both CD3 and CD28 to ensure maximal suppression of immune activation. Interestingly, CD28 downregulation was lost in the first node of the SIVcpz/HIV-1 lineage (Node 51), coinciding with the loss of CD3 downregulation and the acquisition of Vpu. Previous work from our group has shown that both HIV-1 Nef and Vpu independently downregulate CD28 within infected cells (23). While it is unknown whether SIVcpz Vpu downregulates CD28, using the same rationale as with CD3 downregulation, we speculate that Vpu-mediated CD28 downregulation in the SIVcpz/HIV-1 lineage likely reduced the selective pressure on Nef to downregulate CD28 to the same extent as its SIV counterparts that lack Vpu. This could explain why the loss of Nef-mediated CD3 and CD28 downregulation occurred at the same time, which happened to coincide with the acquisition of Vpu.

Our findings show that only the Nef corresponding to the ancestor of all primate lentiviruses, Node 35, could efficiently downregulate CD3 and CD28, while Nefs corresponding to the intermediate nodes (Nodes 51–59) completely lost the ability to downregulate CD3 and displayed only modest improvements in CD28 downregulation over time. These observations strongly align with previous literature on the functional evolution of lentiviral Nef proteins, which assessed the function of extant Nef sequences from different lineages to infer the respective evolutionary history. Combined with our earlier work demonstrating consistent patterns of SERINC5 and CD4 downregulation across the same ancestral Nefs, these findings further validate the accuracy of our bioinformatics pipeline in reconstructing functionally relevant ancestral proteins within a given viral lineage (33). Taken together, our results strongly support the broader application of this pipeline to reconstruct ancestral Nef proteins in other SIV/HIV lineages beyond the SIVcpz/HIV-1 group M lineage to better understand the functional evolution of Nef. More broadly, we propose that this workflow can be extended to other HIV-1 proteins, and even to proteins from unrelated viruses, as a general strategy to explore how viral protein functions evolved—particularly in the context of cross-species transmission events.

### Conclusions

To conclude, our study provides functional evidence that the ability of Nef to downregulate CD3 and CD28 was present in the ancestral primate lentivirus but lost in the lineage giving rise to HIV-1. The corresponding effect on T cell activation further demonstrates the important functional consequences of these Nef functions to the pathogenicity of infection. Together, these results shed light on the evolutionary dynamics of Nef modulation of T cell activation and its impact on the pathogenesis of modern infections.

## MATERIALS AND METHODS

### Ancestral sequence reconstruction

The reconstructed ancestral Nef sequences were generated as described previously (33). Briefly, a representative selection of Nef amino acid sequences from different primate lentiviruses was obtained from the Los Alamos National Laboratory (http://www.hiv.lanl.gov/) and GenBank (https://www.ncbi.nlm.nih.gov/genbank) databases. A multiple sequence alignment was generated using MAFFT (version 7.271) (50), and a maximum-likelihood tree was reconstructed using IQ-TREE (version 1.3.11.1) (51) to serve as a starting tree for Bayesian analysis. BEAST (version 1.10.4) (52) was then used to sample time-scaled phylogenies from the posterior distribution. From the resulting posterior sample of trees, we identified the most frequent trajectory of internal nodes from the root to the common ancestor of HIV-1/M, comprising six internal nodes. Among the *n* = 4,535 trees that followed this maximum support trajectory, we randomly selected 1,000 trees. For each of these trees, we used Historian (version 0.1) (53) to reconstruct the ancestral amino acid sequences at the internal nodes and then generated a consensus sequence across the 1,000 trees. The consensus amino acid sequence at each node was converted to nucleotides using codon distributions from extant sequences, from which the most frequent codon was assigned to each residue (see https://doi.org/10.5281/zenodo.8010261).

### Cell culture

HEK293T cells (ATCC, Manassas, VA, USA) were maintained in Dulbecco’s modified Eagle’s medium containing 4 mM L-glutamine (Cytiva Life Sciences, Vancouver, BC, Canada), 4.5 g/L glucose (Cytiva Life Sciences), and supplemented with 1% 100 μg/mL penicillin-streptomycin (HyClone, Logan, UT, USA) and 10% fetal bovine serum (FBS–Wisent, St. Bruno, QC, Canada). Cell lines were grown at 37°C in the presence of 5% CO_2_ and sub-cultured according to the manufacturer’s instructions.

Primary human PBMCs were isolated from healthy donors, and CD4^+^ T cells were purified using the RosetteSep Human CD4^+^ T cell Enrichment Cocktail (StemCell, Vancouver, BC, Canada) and cryopreserved. Upon thawing, cells were activated with Immunocult Human CD3/CD28/CD2 T cell activator (StemCell) and maintained in Immunocult-XF T cell Expansion Medium (StemCell) supplemented with recombinant human IL-2 (10 ng/mL, ThermoFisher, Whitby, ON, Canada). Primary cells were grown at 37°C in the presence of 5% CO_2_ and sub-cultured for 3 days post-thaw.

### DNA constructs

Previously, the ancestral *nef* sequences were commercially synthesized (GeneArt Gene Synthesis, ThermoFisher) and cloned into pN1 expression vectors (Clontech) expressing Nef C-terminally fused to eGFP (33). For this study, the ancestral *nef* sequences were cloned into replication-incompetent pNL4.3 ΔGag/Pol eGFP ΔVpu Nef proviral plasmids using the pN1 Nef-eGFP expression vectors as templates for PCR amplification (33). Additionally, controls, including NL4.3 Nef, NL4.3 LL_164-165_AA Nef, SIVmac239 Nef, and SIVmac239 I_123_L L_146_F Nef, were also cloned into the pNL4.3 backbone for downstream pseudovirus generation. To improve viral production, the reconstructed Nef amino acid sequences were analyzed using BLASTp to identify related primary Nef sequences (54). For each reconstructed Nef sequence, individual residues and codons were compared to those in the related primary isolates. If the amino acid residue at a given position was conserved, the codon from the primary isolate was substituted into the reconstructed *nef* sequence. This manual codon optimization ensured that codons typical of naturally circulating viral strains were incorporated, with the aim of enhancing viral gene expression and pseudovirion production. This process was performed manually for all reconstructed *nef* sequences. Importantly, the resulting codon-optimized sequences encoded the same amino acid sequences as the original reconstructed Nef sequences (33). The manually optimized *nef* sequences were synthesized using the GeneArt Gene Synthesis service. The proviral plasmid was engineered to readily clone *nef* using flanking 5′ XmaI and 3′ NotI restriction sites as previously described (23). Forward and reverse primers were designed to clone each respective *nef* gene into the proviral backbone by harboring XmaI and NotI restriction sites, respectively. The pCMV-DR8.2 plasmid (Addgene, Waterdown, MA, USA; Cat#12263, gift from Didier Trono) encoding Gag/Pol and the pMD2.G plasmid encoding vesicular stomatitis virus glycoprotein (VSV-G; Addgene, Cat#12259, gift from Didier Trono) were also used for transfections where indicated.

To exogenously express CD3ζ in the absence of the TCR, we used a human CD8α-human CD3ζ (huCD8α- huCD3ζ) fusion construct encoding the extracellular and transmembrane domains of huCD8α (accession: M12824; residues 1–208) fused to the cytoplasmic tail of huCD3ζ (accession: NM_000734; residues 83–194). This was a kind gift from Dr. Frank Kirchhoff (University Clinic of Ulm, Ulm, Germany) and has been previously described (30). To generate fusion vectors expressing CD3ζ from different primates, the nucleotide sequences corresponding to the CD3ζ cytoplasmic domain of rhesus macaques (*Macacca mulatta*; accession: DQ437670), sooty mangabeys (accession: XM012091107), African green monkeys (accession: XM007989658), and Ma’s night monkeys (*Aotus nancymaae*; accession: NM001308517) were first synthesized using the GeneArt Gene Synthesis service. Overlapping PCR mutagenesis was subsequently used to replace the huCD3ζ nucleotide sequence for the CD3ζ cytoplasmic domain of the previously mentioned primate species. In the first PCR reaction, the nucleotide sequence corresponding to the extracellular and transmembrane domains of huCD8α was amplified using a forward primer bearing the XbaI restriction site and a reverse primer with a tail complementary to the 5′ region of the CD3ζ cytoplasmic sequence of the aforementioned primate species. In the second PCR reaction, the nucleotide sequence corresponding to the primate CD3ζ cytoplasmic domain was amplified with a forward primer complementary to the 3′ region of the extracellular/transmembrane domain of huCD8α and a reverse primer harboring the MluI restriction site. In the final PCR reaction, both purified amplicons were mixed 1:1 and amplified using the 5′ forward primer bearing the XbaI restriction site and the 3′ reverse primer bearing the MluI site to generate the huCD8α-CD3ζ construct. This fusion was then cloned into the pCG huCD8α-CD3ζ plasmid accordingly.

To induce the relevant mutations into Node 35 and SIVmac239 Nef, the Q5 Site-Directed Mutagenesis Kit was used according to the manufacturer’s instructions (New England Biolabs, Ipswich, MA, USA). For protein expression analysis, the reconstructed *nef* sequences, along with SIVmac239 *nef* and all corresponding *nef* mutants, were PCR-amplified using sequence-specific forward primers harboring a 5′ EcoRI restriction site and sequence-specific reverse primers harboring a 3′ BamHI restriction site. To generate a plasmid expressing a Nef-eGFP fusion protein, the reverse primer was additionally designed to mutate the *nef* stop codon to a TGT cysteine codon. PCR products were subsequently purified and cloned into the pN1 expression vector (Clontech), which contains the 3′ *egfp* ORF. To clone the Node 35 and SIVmac239 *nef* mutants into the proviral backbone, sequence-specific forward primers harboring a 5′ XmaI restriction site and sequence-specific reverse primers harboring a 3′ NotI restriction site were designed, followed by PCR amplification, purification, and subsequent cloning into the pNL4.3 ΔGagPol eGFP ΔVpu Nef proviral plasmid. All DNA constructs used in this study were verified by Sanger Sequencing at the London Regional Genomics Centre prior to functional analysis.

### Cell surface CD3 downregulation in HEK293T cells

For transfection assays to assess cell surface CD3 downregulation, HEK293T cells were seeded in 6-well plates 24 hours prior to transfection. HEK293T cells were then triply transfected with 1 μg of the cloned pNL4.3 ΔGag/Pol eGFP ΔVpu Nef plasmids, 1 μg of the pCMV-DR8.2 plasmid, and 0.2 μg of the pCG huCD8α-CD3ζ plasmids using PolyJet (FroggaBio, North York, ON, Canada). Forty-eight hours post-transfection, HEK293T cells were washed in 1× phosphate-buffered saline (PBS) without calcium and magnesium (Wisent), lifted with 0.25% Trypsin-EDTA (ThermoFisher), and collected in a 96-well U-bottom plate. Cells were then washed with 1× PBS containing calcium and magnesium (Wisent), stained with Zombie NIR viability dye (BioLegend, San Diego, CA, USA), and subsequently fixed with 1.5% paraformaldehyde (PFA; Electron Microscopy Sciences, Hatfield, PA, USA). Cells were then washed with fluorescence-activated cell sorting (FACS) buffer (3% FBS and 5 mM EDTA in 1× PBS). For cell surface staining of the huCD8α as a proxy for cell surface CD3 levels, cells were stained with 1:25 anti-human CD8α phycoerythrin (PE; clone HITα; BioLegend). The isotype control sample was stained with equivalent amounts of the PE mouse IgG1, κ-Isotype Control Antibody (clone MOPC-21; BioLegend). Finally, cells were washed again in FACS and resuspended in 1× PBS prior to flow cytometry analysis.

### Virus generation

For VSV-G pseudotyped lentivirus production, HEK293T cells were seeded in 6-well plates 24 hours prior to transfection. HEK293T cells were then triply transfected with 0.83 μg of the cloned pNL4.3 ΔGag/Pol eGFP ΔVpu Nef plasmids, 0.83 μg of the pCMV-DR8.2 plasmid, and 0.4 μg of the pMD2.G plasmid encoding VSV-G using PolyJet. Virus-containing supernatants were collected 72 hours post-transfection, supplemented with 20% FBS, filtered using a 0.45 μm filter (ThermoFisher), and stored at −80°C for downstream experiments.

### Viral transduction

For transduction of primary cells, huCD4^+^ T cells 6 days post-thaw were pelleted and resuspended with each VSV-G-pseudotyped virus, 8 μg/mL Polybrene (Sigma-Aldrich, St. Louis, MO, USA), and complete Roswell Park Memorial Institute medium (RPMI) 1640 media—containing L-glutamine and supplemented with 100 μg/mL penicillin-streptomycin, 1% sodium-pyruvate (HyClone), 1% non-essential amino acids (HyClone), and 10% FBS. Subsequently, cells were spinoculated at 2,880 *× g* for 2 hours at room temperature. After spinoculation, cells were resuspended in complete RPMI and IL-2 (10 ng/mL) for an additional 48 hours.

### Cell surface CD3 and CD28 downregulation in primary cells

For cell surface staining of huCD4^+^ T cells 48 hours post-transduction, cells were collected and washed with 1× PBS and stained with Zombie NIR viability dye. Cells were then fixed with 1% PFA and washed in FACS buffer. For cell surface staining of CD3 and CD28, 1:80 anti-human CD3 PE (clone UCHT1; BioLegend) and 1:25 anti-human CD28 APC (clone CD28.2; BioLegend) were used. Isotype control samples were stained with PE mouse IgG1, κ-Isotype Control Antibody (clone MOPC-21; BioLegend) and APC mouse IgG1, κ-Isotype Control Antibody (clone MOPC-21; BioLegend). Finally, cells were washed again in FACS and resuspended in 1× PBS prior to analysis.

### T cell activation and intracellular cytokine analysis

To assess T cell activation, huCD4^+^ T cells were cultured and transduced as described above. Forty-eight hours post-transduction, they were re-activated with Immunocult Human CD3/CD28/CD2 T cell activator. Cells were then incubated for an additional 24 hours before being stained with Zombie NIR viability dye, fixed with 1% PFA, and stained with 1:20 anti-human CD69 BrilliantViolet421 (BV421; clone FN50; BioLegend) and 1:200 anti-human CD25 APC (clone BC96; BioLegend) for flow cytometry analysis. Isotype control samples were stained with BV421 mouse IgG1, κ Isotype Ctrl Antibody (clone MOPC-21; BioLegend) and APC mouse IgG1, κ-Isotype Control Antibody (clone MOPC-21; BioLegend). For intracellular cytokine staining, cells were transduced and re-stimulated as described previously, with the addition of 1:1,000 Brefeldin A (BioLegend) 8 hours post-re-activation to inhibit cytokine secretion. Cells were stained with Zombie NIR viability dye, fixed with 1% PFA, and washed with FACS buffer. Cells were then permeabilized with Perm/Wash Buffer (BD Biosciences, Franklin Lakes, NJ, USA) and incubated with Human TruStain FcX blocking solution (BioLegend) to prevent unwanted antibody binding. Cells were then stained with 1:20 anti-human IL-2 PE (clone MQ1-17H12; BioLegend) and 1:20 anti-human IFNγ AlexaFluor647 (AF647; clone 4S.B3; BioLegend). Isotype control samples were stained with PE rat IgG2α, κ Isotype Ctrl Antibody (clone RTK2758; BioLegend) and AF647 Mouse IgG1, κ Isotype Ctrl (ICFC) Antibody (clone MOPC-21; BioLegend). Cells were then washed with Perm/Wash buffer and FACS and resuspended in 1× PBS prior to analysis.

### Flow cytometry analysis

For flow cytometry analysis of the HEK293T transfection experiments, cells were analyzed with the BD Biosciences LSR-II cytometer. For flow cytometry analysis of all other experiments, cells were analyzed with the Beckman Coulter CytoFLEX S (Brea, CA, USA). Both cytometers are located at the London Regional Flow Cytometry Facility (London, ON, Canada). Flow cytometry data were analyzed using FlowJo software (version 10.10.0, FlowJo LLC, Ashland, OR, USA).

### Data and statistical analysis

Relative levels of cell surface receptors and intracellular expression of cytokines were determined by quantifying geometric mean fluorescence intensity (gMFI) of the respective fluorophores after gating on single, live (Zombie NIR^-^), and transfected/transduced cells (eGFP^+^), as illustrated in Fig. 1B. For the analysis of cell surface huCD8α-CD3ζ levels, Nef-mediated CD3 downregulation was calculated as a percentage of SIVmac239 wild-type Nef by dividing the gMFI of PE for SIVmac239 WT Nef by the gMFI of PE for each Nef sample, and then multiplying by 100.


$$CD3\;downregulation\;(\%\;of\;SIVmac239\;WT\;Nef)=\left(\frac{gMFI\;PE\;SIVmac239\;WT\;Nef}{gMFI\;PE\;Nef\;sample}\right)\times 100$$

In primary cells, fold CD3/CD28 downregulation was calculated by dividing the gMFI of PE/APC for ΔNef by the gMFI of PE/APC for each Nef sample, such that fold downregulation by ΔNef was equal to 1.0.


$$\text{Fold CD3 downregulation}\left(relative\;to\;\Delta Nef\right)=\frac{gMFI\;PE\vee APC\;\Delta Nef}{gMFI\;PE\vee APC\;Nef\;sample}$$

For the relative expression of cell surface CD69/CD25, this was calculated by dividing the gMFI of PE/APC for each Nef sample by the gMFI of PE/APC for ΔNef, such that relative expression by ΔNef was equal to 1.0.


$$\text{CD69 or CD25 expression}\left(relative\;to\;\Delta Nef\right)=\frac{gMFI\;PE\vee APC\;Nef\;sample}{gMFI\;PE\vee APC\;\Delta Nef}$$

This was also completed for the relative expression of IL-2/IFNγ by extracting the gMFI of PE/AF647, such that relative expression by ΔNef was equal to 1.0.


$$IL\text{-}2\;or\;IFN\gamma \;expression\;(relative\;to\;\Delta Nef)=\frac{gMFI\;PE\vee AF647\;Nef\;sample}{gMFI\;PE\vee AF647\;\Delta Nef}$$

One-way ANOVA was used to test for significant variation among groups and Tukey’s honestly significant difference test to identify significant differences between specific pairs of groups while adjusting for multiple comparisons. For all statistical tests, *P* values less than or equal to 0.05 were considered statistically significant. Statistical tests and figures were generated using GraphPad Prism (Version 8, Boston, MA, USA).

### Western blot analysis

To determine Nef-eGFP fusion protein expression levels, HEK293T cells were seeded in 6-well plates 24 hours prior to transfection. HEK293T cells were then transfected with 1 μg of the cloned pN1 Nef-eGFP plasmids using PolyJet. Twenty-four hours post-transfection, the cells were washed once with cold 1× PBS, then lysed by rocking in lysis buffer (0.5 M HEPES, 1.25 M NaCl, 1 M MgCl_2_, 0.25 M EDTA, 0.1% Triton X-100, and 1× complete Protease Inhibitor Tablets [Roche, Indianapolis, IN, USA]) for 1 hour at 4°C. Supernatants were then clarified by spinning at 20,000 × *g* for 30 minutes at 4°C, mixed with 5× SDS-PAGE sample buffer (0.3 M Tris, pH 6.8, 2.8 M β-mercaptoethanol, 0.5% bromophenol blue, 50% glycerol, and 10% SDS), and boiled at 98°C for 8 minutes. Samples were run on 12% SDS-PAGE gels, followed by transferring to nitrocellulose membranes (Cytiva; Cat# 10600002). Membranes were blocked with 5% milk in TBST (50 mM Tris, 150 mM NaCl, and 0.1% Tween 20) for 1 hour at room temperature, followed by overnight incubation at 4°C with the appropriate primary antibody in 5% milk in TBST. The following primary antibodies were used: 1:5,000 mouse anti-GFP monoclonal antibody (ThermoFisher; Cat# MA5-15256) and 1:20,000 mouse anti-GAPDH monoclonal antibody (ThermoFisher; Cat#AM4300). Membranes were washed three times with TBST and incubated for 2 hours at room temperature with 1:20,000 (for GAPDH detection) and 1:5,000 (Nef-eGFP detection) of the HRP-conjugated goat anti-mouse IgG (H + L) secondary antibody (ThermoFisher; Cat#31430) in 5% milk in TBST. Blots were subsequently washed in TBST and developed using Immobilon Classico Western HRP Substrate (Millipore; Cat#WBLUC0500) and imaged using a C-DiGit chemiluminescence Western blot scanner (LI-COR Biosciences, Lincoln, NE, USA).

## Data Availability

The accession numbers of the codon-optimized ancestral reconstructed *nef* nucleotide sequences corresponding to Nodes 35, 51, 52, 55, 56, and 59 utilized within this study, deposited in GenBank, are PX136282 to PX136287.

## SUPPLEMENTAL MATERIAL

Supplemental figures jvi.01548-25-s0001.pdf Figures S1 to S3.

ASM does not own the copyrights to Supplemental Material that may be linked to, or accessed through, an article. The authors have granted ASM a non-exclusive, world-wide license to publish the Supplemental Material files. Please contact the corresponding author directly for reuse.
