# Supplementary material for: Ancestral reconstruction supports that loss of Nef-mediated T cell modulation coincided with the emergence of pathogenic lentiviruses
Source: J Virol. 2025 Dec 2;99(12):e01548-25. doi: 10.1128/jvi.01548-25 (PMC12724223; doi:10.1128/jvi.01548-25)
Supplement: Supplemental figures — Figures S1 to S3. [file jvi.01548-25-s0001.pdf]

Supplemental Material

Ancestral reconstruction supports that loss of Nef-mediated T cell modulation coincided with the emergence of pathogenic lentiviruses

Angelina M. Baldino[a], Mitchell J. Mumby[a], Cassandra R. Edgar[a], Abayomi S. Olabode[b], Art F.Y. Poon[a,b,c], Jimmy D. Dikeakos[a]#

[a]Department of Microbiology & Immunology, Western University, London, Canada

[b]Department of Pathology & Laboratory Medicine, Western University, London, Canada

[c]Department of Computer Science, Western University, London, Canada

A

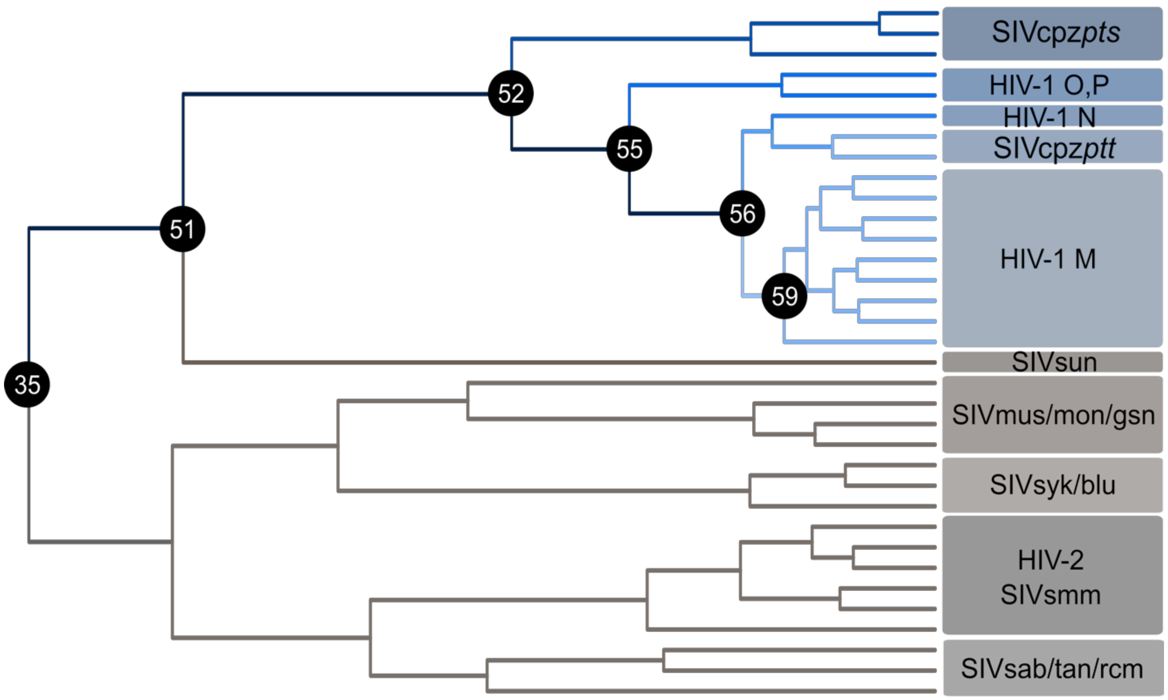

Figure S1: Reconstructed phylogeny containing ancestral Nef sequences. (A) Maximum clade credibility tree (MCC) of 1,000 BEAST trees with consistent nodes used to obtain ancestral Nef sequences.[1]

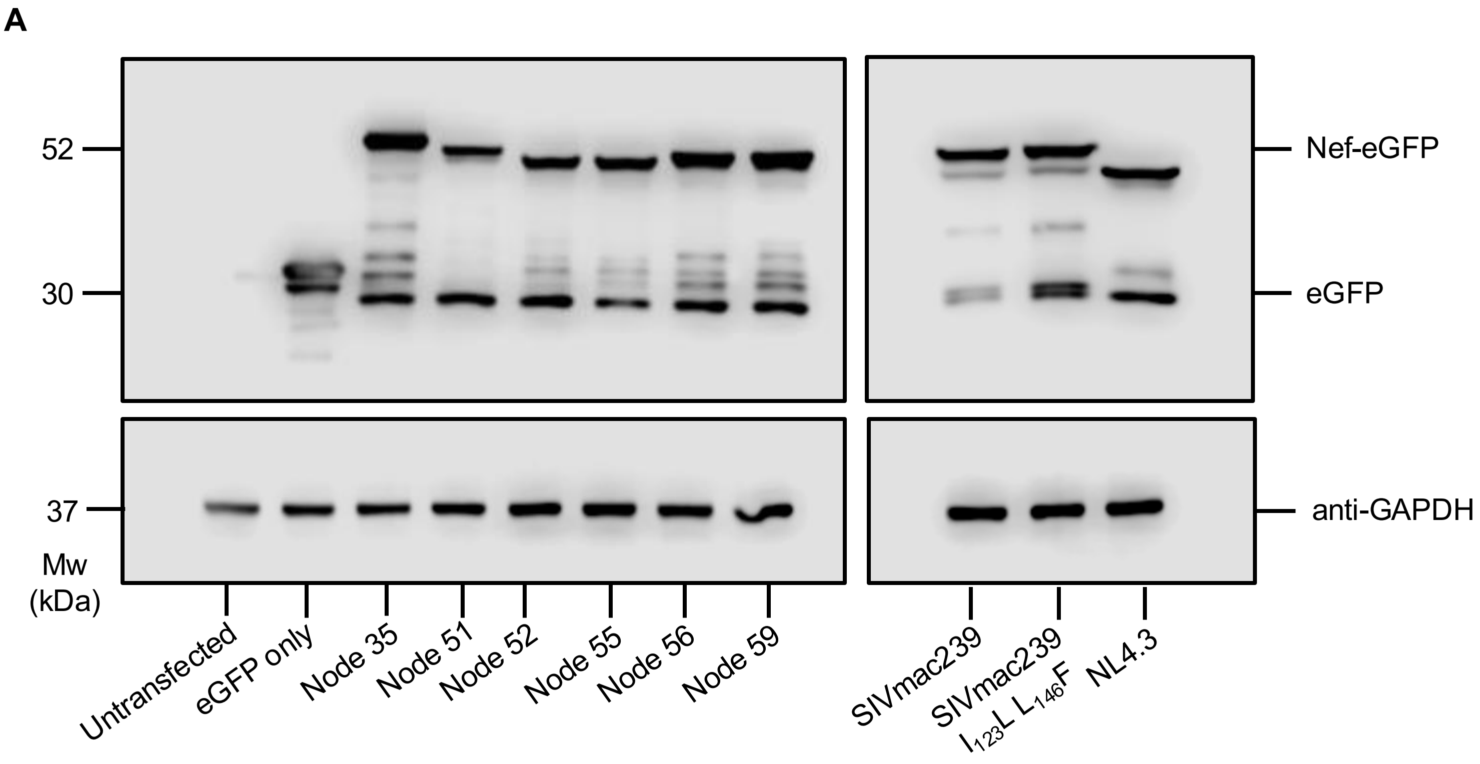

Figure S2: Evaluation of ancestral Nef-eGFP protein expression. Lysates were collected from HEK293T cells 24 hours following transfection with cloned pN1 Nef-eGFP fusion vectors. Nef-eGFP fusion proteins were detected from three independent experiments (n = 3). **(A)** Representative Western blot illustrating the expression of Nef-eGFP fusion proteins or eGFP alone, with the approximate molecular weight values in kDa indicated accordingly. kDa: kilodalton; GAPDH: glyceraldehyde-3-phosphate dehydrogenase; eGFP: enhanced green fluorescent protein

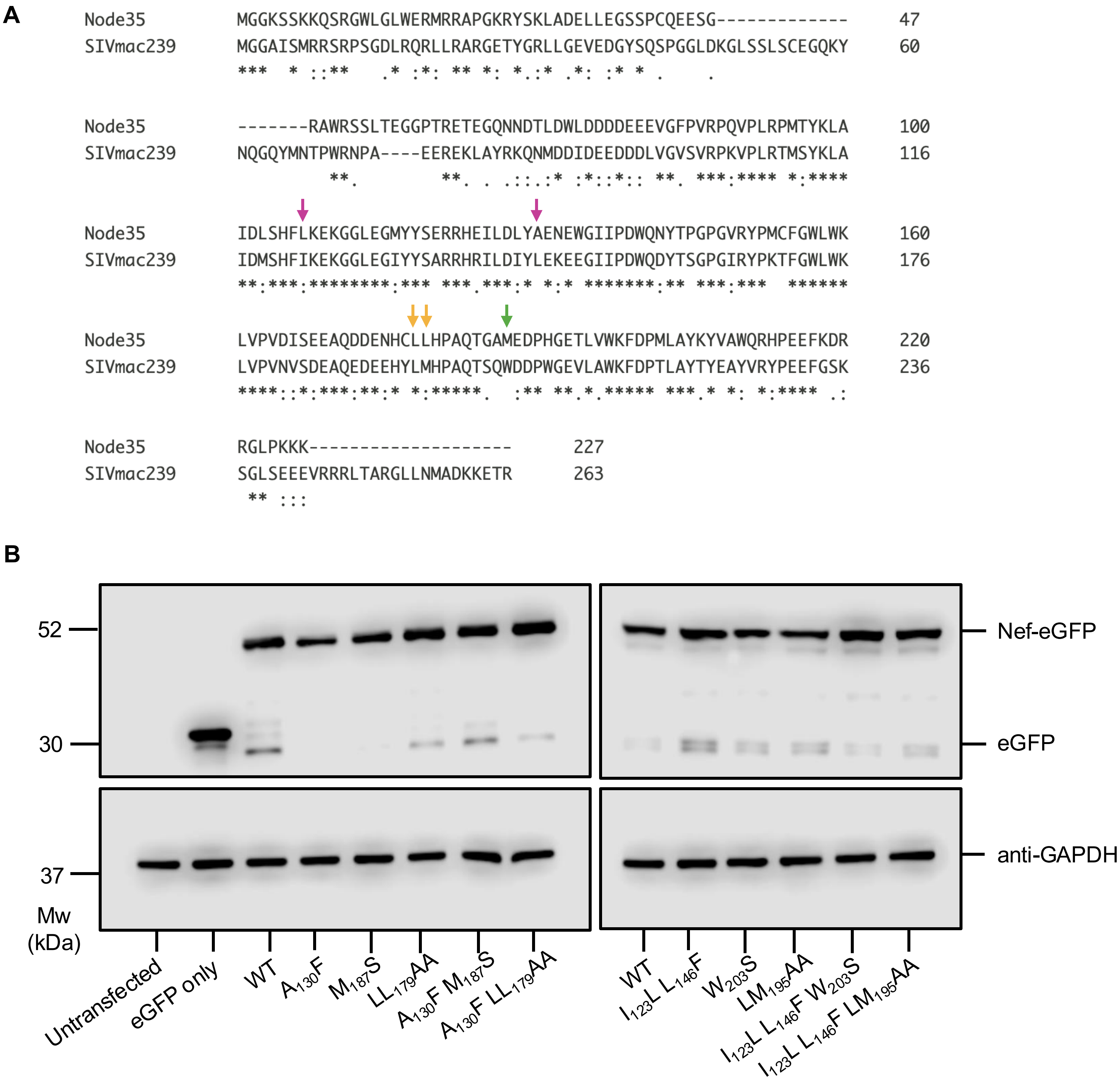

Figure S3: Generation of Node 35 Nef and SIVmac239 Nef mutants. (A) Amino acid sequence alignment of Node 35 and SIVmac239 Nef. Arrows represent residues mutated (as displayed in Fig 5A) including mutations in the hydrophobic core (A_{130} in Node 35 and I_{123} L_{146} in SIVmac239 Nef; pink arrows), dileucine motif (LL_{179} in Node 35 and LM_{195} in SIVmac239 Nef; yellow arrows), and M_{187} in Node 35 and W_{203} in SIVmac239 Nef (green arrow). **(B)** Evaluation of Node 35 Nef-eGFP and SIVmac239 Nef-eGFP protein expression. Lysates were collected from HEK293T cells 24 hours following transfection with cloned pN1 Nef-eGFP fusion vectors. Nef-eGFP fusion proteins were detected from three independent experiments (n = 3), with (B) displaying a representative Western blot for the expression of Nef-eGFP fusion proteins or eGFP alone, with the approximate molecular weight values in kDa indicated accordingly. kDa: kilodalton; GAPDH: glyceraldehyde-3-phosphate dehydrogenase; eGFP: enhanced green fluorescent protein

References

(1) Olabode, A. S.; Mumby, M. J.; Wild, T. A.; Muñoz-Baena, L.; Dikeakos, J. D.; Poon, A. F. Y. Phylogenetic Reconstruction and Functional Characterization of the Ancestral Nef Protein of Primate Lentiviruses. *Mol Biol Evol* **2023**, *40* (8), msad164. https://doi.org/10.1093/molbev/msad164
